# Essential Nature of Caries. No. 1

**Published:** 1867-09

**Authors:** H. R. Noel

**Affiliations:** Professor of Physiology, Baltimore College of Dental Surgery.


### AMERICAN JOURNAL

OF

### DENTAL SCIENCE.

Vol. I. THIRD SERIES.—SEPTEMBER, 1867. No. 5.

### ORIGINAL COMMUNICATIONS.

### ARTICLE I.

### *Essential Nature of Caries. No. 1.*

By H. R. Noel, M. D., Professor of Physiology,
Baltimore College of Dental Surgery.

Separation being true prophylaxis, what is the "Essential Nature of Caries"? Is it a vital process intrinsically? Is it a physical or chemical process intrinsically? Is it a blending of the two? We shall endeavor to answer these questions, and elicit the physiological explanation of the facts given us by observation and practical experience.

To fully grasp this subject, it is necessary that we have clear ideas of the minute anatomy of the teeth; but the limits of this article preclude an elaborate discussion of their histology. We shall however give a brief but comprehensive description of their microscopic structure, both as regards views formerly entertained, and views more recently advocated, as results of the most rigid investigations with the highest powers of the microscope. These views are widely and essentially different, and we would here suggest a close and careful attention to each, as we produce them. We shall assume much as already known, and give only the veriest essentials in our descriptions. But even then, we shall be compelled to introduce much that is uninteresting except to the thorough student, dry details of microscopic appearances, as given by different observers.

General character of Dentine, Enamel, and Cementum.

1. Dentine consists of microscopic tubules, opening by their larger ends into the cavity of the pulp, and radiating in lines perpendicular to the walls of this cavity towards the periphery of the tooth. The diameter of these tubular rods at their larger ends is 1.4500th with a bore of 1.10000th of an inch; at the peripheral ends, which exhibit numerous branches, they are immeasurably fine. Intertubular substance faintly granular.

2. Enamel prismatic *solid* fibres, so closely packed, that there is no intervening material; prisms four or six sided, adhering by one end to dentine and free by the other; 1.5600th inch in diameter.

3. Cementum has characters of true bone.*

1. Dentine.—Every part of the surface of the pulp cavity is pierced by microscopic pores, about 1.10000th of an inch in diameter, which are orifices of tubes radiating from the pulp cavity to the periphery of the dentine, with a general direction vertical to that surface. These are the dentinal tubes.

Thin sections of dentine taken across the tubes viewed by transmitted light with a five hundred times linear magnifying power, show the clear area of the tube more or less occupied by a dark, sub-granular, opaque calcareous substance; and a clear border to the area, neatly defined from the intertubular substance, and indicating the proper parietes of the tube (i. e. marking the distinct outline of calibre and walls, tube and intertubular substance.)

"If the tubes were described according to the order of their formation, the *peripheral extremitics* would be their *beginnings*, and the dichotomous

---

*Syllabus of Lectures on Physiology. By Prof. J. C. Ceabell. W. Va., 1853.

*divisions* would rather be the *anastomores of the more numerous and smaller tubes* as they gradually converge to terminate by *fewer and wider extremities*, in the pulp cavity." Odontography pp. 459—60—61. Richard Owen.

2. "Enamel of the human teeth consists of long and slender solid prismatic, for the most part hexagonal, fibres of phosphate, carbonate and fluate of lime."

"Each fibre is 1.5000th of an inch in thickness, and is marked throughout its entire course by faint, close set transverse striæ."—Idem pp. 464 and 465.

3. "Cementum.—This third substance is thinnest upon the crown, and very gradually increases in thickness as it approaches the end of the fang; it is only on the implanted part of the tooth that the radiated cells which demonstrate the close analogy between cementum and bone exist: elsewhere the clear basis of cement above is present, and this is soon worn away from the enamel of the crown. There are no vascular canals in human cement except where it happens to be abnormally thickened. The chemical composition of both the animal basis and earthy salts of this dental tissue and of bone are identical. Cementum cells oblong, circular, fusiform, have an average diameter of 1-2000th of an inch: and the fine radiating tubes diameter 1-2009th of an inch.—*Idem* 466 and '67.

1.—"Dentine is made up of two distinct parts, dentinal tubes, and an intertubular tissue. The course of the tubes bending, curving, undulating, branching, anastomosing, &c., as they approach the enamel, have short, transverse, dilatations at tolerably regular intervals."—*Tomes' Dental Phy. and Surgy.*, pp. 32 and 34.

"When measured by the micrometer, the internal diameter of the largest tube is about 1-100000th of an inch; and diameter including parieties 3-10000th of an inch."—*Idem* p. 39.

Mr. Tomes' diagrams show distinctly the outlines of calibre or bore, walls, tubes and intertubular substance corresponding with Mr. Owen in every particular.

2.—"Enamel is composed of semi-transparent fibres placed side by side and closely united. Their form is an approximation to a six-sided prism, and their size tolerably uniform, being from the 2.1000th to the 3.10000th of an inch in diameter."—*Idem p.* 53.

Dentinal tubes may be sometimes prolonged into the enamel, and the fibre may then be hollow instead of solid, but this is rarely found in adult teeth.

3.—"Cementum.—The general description of the structure is equally applicable to tooth bone and osseous tissue."—*Idem* p. 58.

The above views are those which obtained for many years, but we shall find that they are in some respects radically erroneous. Mr. Tomes in a more recent work, "Dental Surgery," has so far modified and changed his views as regards the microscopic characters of dentine, that some opinions formerly supported, are now utterly abandoned. He still acknowledges the solid character or prismatic form of the enamel rods, columns or fibres and their cross striæ. As regards the dentinal tubes, there is a most important and radical change, for instead of open tubules as before described, he has now a description, clear and forcible, plain and satisfactory, of soft, fleshly fibrillæ running from the pulp to these tubes, filling these tubes, and ramifying as the tubes do. There are therefore, in the dentine in a perfect state of health, as when the tooth occupies the normal relations in the mouth, *no open tubes bearing fluids.* But we have fleshly fibrillæ running from the peripheral surface of the dentine back to the pulp, and these fibrillæ are vitally and organically continuous with the intimate structure of the pulp proper.

The descriptions of the tubes, their dichotomous divisions, anostomoses, branchings, &c., answer equally as well and are as perfectly accurate as regards the fibrillæ in question. We should not say, that the pulp tissue proper sends out prolongations of soft, solid or semi-solid fibrillæ, which fill the spaces formerly supposed to be tubes in the dentine, and extend to their ultimate ramifications, and their minutest subdivisions; but beginning at the point, the periphery, *we would rather say*, these fibrillæ run from *periphery* to *pulp*, and in this we agree fully with Richard Owen as regards the formation, of what he terms the tubes. As we proceed this idea will be more clearly developed. There are no tubes in dentine—so far as we are able to ascertain by the present powers of the microscope, and though this may seem a rash assertion, we hope to be able to demonstrate its truth.

Mr. Tomes has succeeded in making many preparations, that do seem to absolutely demonstrate the non-existence of tubes, but invariable presence of fibrillæ in the dentine. Dr. L. S. Beale has also specimens, which he thinks, substantiate Mr. Tomes' statements.

So far as our microscopic knowledge extends, these statements cannot be, or at least have not been refuted, and are acknowledged by our best authorities to be absolutely true.

The presence of these fibrillæ modifies our physiological views as to the structure of the teeth, their vitality, liability to changes, &c. There is a point of some interest here —it is one mentioned by Mr. Tomes, and regard the microscopic appearance of a piece of carious dentine.

"Under the microscope the section looks as though it might have been built up of multitudes of *tobacco-pipe stems*, united by an intervening substance."—*Dental Surg.*, p. 358.

The intervening substance was *decaying first*, and not the walls of the so-called tubes, or the fibrillæ.

"As regards enamel the *central portion* of the rods gave way first, or suffered first from caries."—*Idem* p. 356.

The point of interest is one to be remembered, as we shall have occasion to refer to it again, and also to use it in an argument against the vital theory of caries.

The intertubular substance of dentine, and the centres of the enamel rods first become carious.

As regards the developement of the different structures, we shall assume, that the generally received opinions are perfectly familiar to our readers ; and as we have too little space, to review the opinions of Raschkow, Kolliker, Sent, Nasmyth, Huxley, &c., we shall give a concise and brief notice of the subject, following Tomes and L. S. Beale as the latest and best authorities. There are certain ideas connected with the developement, which believing to be utterly false, we shall ignore, and if referred to at all, it will be in a very casual manner. A careful study of both Tomes' and Beale's works seems to give the following as correct.

The dental pulp is not itself calcified. The performative membrane of Raschkow, Huxley, &c., and the membrane of Nasmyth are one and the same, belonging to the cementum ; and it appears from direct microscopic examination, to be but the uncalcified remains of a membrane that served in the production of the cementum. Both dentine and enamel are formed from elongated, nucleated bodies which have been spoken of as cells, and look like nucleated, columnar epithelium. This is in direct opposition to Huxley's views.

"Cylindrical columns having one or more nuclei, with a certain number of spherical or ovoid nucleated cells bathed in fluid, will be seen to make up this gelatinous matter. Again if the inner surface of the sac be examined by removing a small piece and placing it in fluid in the field of the microscope, we shall see that minute columns similar to those found in the gelatinous substance cover the surface. This is the enamel organ or enamel pulp, from which the columns found in the gelatinous substance have been detached."—Dental Surg. pp. 307 and 308

These are between the internal surface of the sac, and the external surface of the papilla, or coronal surface of the young tooth. Kolliker states:

"The dental sacs consist of consecutive tissue, in which vessels and nerves are distributed; from their base proceeds the dental pulp, which in form resembles the tooth to which it belongs, and consists of an internal portion rich in vessels and eventually in nerves also, and a nonvascular external portion. The latter is bounded by a delicate structureless membrane, the membrana preformativa (Raschkow,) which has no further relation to the developements of the tooth. Beneath this lie cells of 0.016 to 0.024''' in length, and 0.002 to 0.0045 in breadth, with very beautiful vesicular nuclei, and distinct single or multiple nucleoli; they are arranged close together over the whole surface at the pulp, like an epithelium, though not so sharply defined internally as it would be, but gradually passing, at least apparently by smaller cells into the parenchyma."

We see the unity of description here and the clearness of it, leaves no doubt that these three observers (Kolliker, Tomes, Beale,) are correct upon their main point, i. e. the presence of columnar cells, as entering invariably into the formation of dentine and enamel. The membrane described by Kolliker has no actual existence as a distinct structure, but the elongated nucleated cells, or bodies, are mentioned by all three, they are therefore a common element of description.

Dr. L. S. Beale makes some very instructive statements and throws a great deal of light upon those obscure changes going on between the sac of the tooth and papilla or dental pulp. The changes occurring here are consummated in the production of enamel and dentine, and both are produced from columnar cells, fibres, rods or bodies, by calcification; the cells or rods, &c., spring from the dental pulp and from the walls of the dental sac alike, hence they must meet upon some half way ground. The nucleated rods, that are to form dentine, obtain nutrient plasma from the pulp ; the rods or cells for the enamel obtain the plasma from vessels ramifying on the walls of the sac, and would appear to approach each other as they develope, until the line of demarcation between enamel and dentine is reached. The calcification of the nucleated bodies, rods &c., gives the true enamel and true dentine.

"Before any dentine is formed, and immediately beneath the dentine last formed at all ages, between it and the vascular and nervous pulp is a layer of what looks something like columnar epithelium."

"This tissue consists of elongated, cylindrical bodies, or cells with nuclei, placed at right angles to the outer surface of the pulp. Although the pulp diminishes in size as the formation of dentine proceeds, the pulp does not become dentine ; but this dentine results from changes occurring in a tissue which lies upon the surface of the pulp. This consists of cells like columnar cells. These elongated cells are not separated from the nerves, vessels and connective tissue of the pulp by a demonstrable basement membrane, but their extremities pass amongst the fibres of the connective tissue, and in some instances seem to be continuous with them. It is possible that some may be continuous with the so-called connective tissue corpuscles. As new dentine is formed these cells encroach upon the pulp, the constituent tissues of which gradually diminish in amount." —Structure, Growth and Life, pp. 182, 183.

We notice here that Beale denies the existence of a demonstrable basement membrane between the pulp proper and the "columnar cells," though Kolliker asserts its existence, they agree as to the existence of these cells, bodies, or rods ; Tomes favors the view advocated by Beale and the weight of evidence is certainly in their favor. The organic continuity between these cells and the tissue of pulp proper, throws a light upon the significant fact of the organic continuity of the fibrillæ of the dentinal tubes and pulp tissue. We shall endeavor to show, the direct bearing of this fact, when we discuss the properties of these fibrils or fibrillæ. Between the pulp and dentine already formed, there are at all ages and times, some of these "cells" or columnar bodies, and we find that dentine is being constantly though slowly formed, and the pulp cavity therefore diminishing. The greatest amount of vital change and transformation is therefore between the pulp and dentine ; and we shall also find these or similar "cells" between the cementum formed and membrane investing, hence cementum may increase in amount also. Does caries ever occur at these points?

The elongated bodies, cells, or rods, are the structural elements, that calcified, become dentine and enamel. Those that are to become dentine have a direct organic continuity with the tissues of the pulp, and are nourished from the vessels of the pulp. Those that are to form enamel have a direct organic continuity with the internal vascular surface of the membrane lining the tooth sac, and are nourished from the vessels of this lining membrane. These "cells or prisms," "nucleated bodies or rods" are first formed and then the deposition of inorganic matter takes place, and calcification is the result. The first step is purely vital,—the second probably chemical, or at best, only chemico-vital. These "cells," "rods" "prisms" or "nucleated bodies," (as various writers have thought proper to name the same anatomical element to suit themselves, i. e. these structures between papilla and sac, and connected with both) are undoubtedly vitalized structures, but when once thoroughly calcified, we would respectfully ask are they still vitalized? This is a delicate point and shall be thoroughly discussed.

When calcification takes place the first portion of the "cells" calcified, are those ends where enamel and dentine unite. The "cells" that are to form dentine, calcify at their peripheral ends first, and the calcification gradually extends towards the pulp; on the other hand those cells that are to form enamel, begin to calcify exactly on the line of union between dentine and enamel, but here the calcification extends outwards and towards the vascular membrane lining the tooth sac. Commencing at a common point the process in the two cases proceeds in exactly opposite directions. The last formed portions of enamel are the peripheral ends of the "rods," upon the crown and sides of the tooth. The last formed portions of the dentine are those rods or ends next to the pulp, and the portion next to the fibrils or fibrillæ, i. e. the internal surface of the so-called walls, as seen in the dry tooth. In fact dentine is being formed and fibrils are diminishing in size, pulp cavity diminishing size throughout the life of the tooth.

The "cells" or "elongated bodies," in the case of enamels, begin to calcify first on the exterior of the cell, or what might be called the cell-wall, and the calcification extends into the centre of each prism, but the centres calcify more slowly than the exterior or outer portion of the rod.

(To be Continued.)

